# Volcanic tephra deposition dataset based on interpolated field measurements following the 2021 Tajogaite Eruption on La Palma, Canary Islands, Spain

**DOI:** 10.1016/j.dib.2023.109949

**Published:** 2023-12-12

**Authors:** Christopher Shatto, Frank Weiser, Anna Walentowitz, Reinhold Stahlmann, Samip Shrestha, María Guerrero-Campos, Félix Manuel Medina, Manuel Nogales, Anke Jentsch, Carl Beierkuhnlein

**Affiliations:** a Department of Biogeography, University of Bayreuth, Universitätsstraße 30, 95447 Bayreuth, Germany; b German Aerospace Center (DLR), German Remote Sensing Data Center (DFD), Oberpfaffenhofen, 82234 Wessling, Germany; c Área de Medio Ambiente, Gestión y Planeamiento Territorial y Ambiental (Gesplan S. A.), Avenida 3 de mayo, 71, Tenerife, Canary Islands, Spain; d Consejería de Medio Ambiente, Cabildo Insular de La Palma, Avenida Los Indianos, 20, 38700 Santa Cruz de La Palma, Canary Islands, Spain; e Island Ecology and Evolution Research Group, Institute of Natural Products and Agrobiology (IPNA-CSIC), Avenida Astrofísico Francisco Sánchez 3, 38206 San Cristóbal de La Laguna, Tenerife, Canary Isalnds, Spain; f Disturbance Ecology, University of Bayreuth, Universitätsstraße 30, 95447 Bayreuth, Germany; g Bayreuth Center for Ecology and Environmental Science BayCEER, Universitätsstraße 30, 95447 Bayreuth, Germany; h Geographical Institute Bayreuth, GIB, Universitätsstraße 30, 95447 Bayreuth, Germany; i Departamento de Botánica, Universidad de Granada, Campus Universitario de Cartuja, 18071 Granada, Spain

**Keywords:** Disturbance, Interpolation, Oceanic island, Pyroclastic ash, Volcanic eruption

## Abstract

In 2021, the Tajogaite Volcano erupted along the western slope of the Cumbre Vieja on the island of La Palma, Canary Islands, Spain. Volcanic tephra blanketed a substantial proportion of the island. By our estimations, approximately 23,000,000 m^3^ of pyroclastic ashes and more coarse-grained particles were deposited unto La Palma's land surface in addition to the lava flow. Five months following the initial eruption, we measured the depth of the new ash layer across the island. We combined this data with drone-based observations to compile a dataset comprising the point distribution of ash depth. A spatial interpolation was then performed using Inverse Distance Weighting (IDW) to estimate the ash depth across the island at a 2 m spatial resolution. The interpolation performed well, yielding a root mean squared error (RMSE) value of 0.34 and thus, the dataset offers immense reuse potential for spatial inquiries related to evolutionary traits, vegetation patterns, and vegetation response to disturbance on oceanic islands. In addition, the data can be used to test different spatial interpolation techniques in an effort to improve the accuracy achieved using IDW.

Specifications Table

**Table:** 

Subject	Earth-Surface Processes / Stratigraphy / Geoecology
Specific subject area	*Geostatistical modelling (interpolation) of volcanic ash and tephra deposition using Inverse Distance Weighting (IDW).*
Data format	Raw data
Type of data	Figure, Table
Data collection	A total of 415 in-situ field measurements were sampled across the Island of La Palma using a spade and measuring tape. Holes were dug until the previous soil surface (oxidized discoloration) or bedrock was reached. Holes were dug to a maximum of 150 cm for safety precaution and as many holes were dug at this maximum depth as possible to improve the accuracy of the interpolation. The coordinates of each measurement were noted for subsequent compilation of the shapefile product. Close to the crater, where ash depth exceeded 150 cm, the difference between a digital surface model from drone based structure from motion photogrammetry published by Civico et al. [11] and a 2 m Digital Terrain Model (DTM) from CNIG [12] was calculated. These 66 surface change values were used to accurately complete the tephra depth training dataset in areas not suitable for in-situ sampling. An interpolation was calculated for the entirety of the 481 points in the dataset using the Inverse Distance Weighting (IDW) interpolation approach. The interpolation yielded tephra depth at 2 m spatial resolution for the extent of the terrestrial surface of La Palma. This was exported in GeoTIFF format.
Data source location	Island of La Palma (-18.0°E to -17.7°E, 28.44°N to 28.86°N)
Data accessibility	Repository name: **IDW interpolation dataset of tephra deposition following the 2021 Tajogaite volcanic eruption on La Palma, Canary Islands, Spain** Data identification number: 10.5281/zenodo.8338991 Direct URL to data: https://doi.org/10.5281/zenodo.8338991
Related research article	Weiser, F., Walentowitz, A., Baumann, E., Shatto, C., Guerrero-Campos, M., Jentsch, A., Nogales, M., Medina, F. M., & Beierkuhnlein, C. Combining in-situ monitoring and remote sensing to detect spatial patterns of volcanic sulphur impact on pine needles. *Forest Ecology and Management 549*. https://doi.org/10.1016/j.foreco.2023.121468

## 1 Value of the Data


 

• This dataset is valuable to researchers keen on exploring the effects of the 2021 Tajogaite eruption (La Palma, Canary Islands, Spain) on successional trajectories of the vegetation, changes in topography, soil formation, and selective evolutionary processes.
• This dataset is constructed using deterministic interpolation and demonstrates the utility of using spatial interpolation techniques to estimate tephra deposition processes. Our estimation of the total tephra volume across the terrestrial extent of La Palma was on par with more sophisticated methods [1]. This contributes to a novel field of research: pyroclastic deposits and sedimentation estimation.
• Researchers may use this dataset to explore fundamental evolutionary processes unique to oceanic islands, where volcanic activity is the primary disturbance regime, by comparing this case study with processes on other oceanic islands of the world.
• The dataset is suitable for monitoring changes to water availability and subsequent changes to the local water cycle due to ash deposition and investigate how these influence surrounding vegetation and freshwater provision to the local human population.

## 2 Objective

**Fig. 1:**
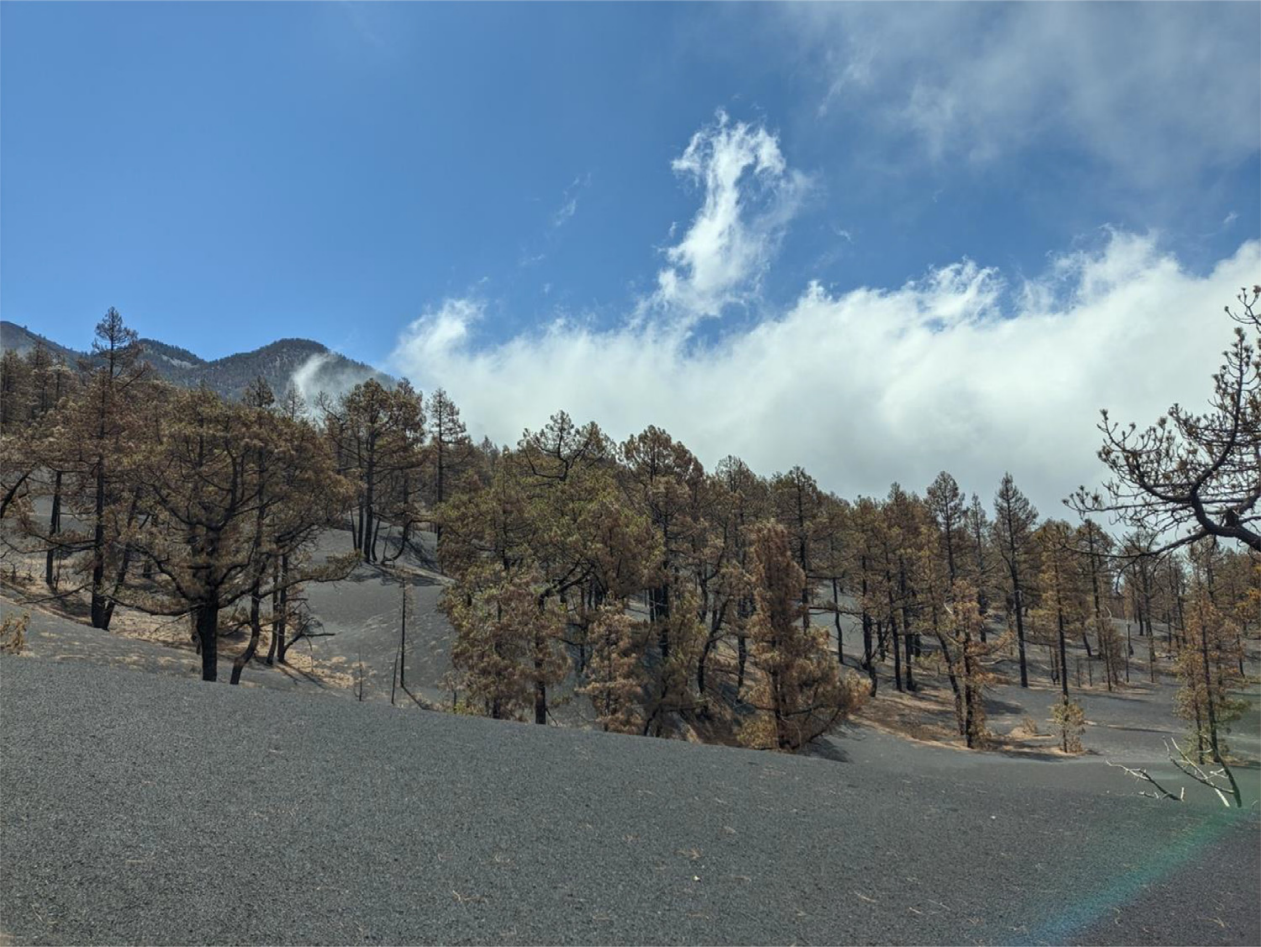
Canary Pine forest close to the eruption crater, showing chlorotic damage to the trees caused by sulfuric gases and the thick layer of deposited tephra covering the entire previous understory (Photo: F. Weiser).

Volcanic eruptions have a strong impact on surrounding ecosystems. The recent eruption of the Tajogaite volcano on the island of La Palma, Spain, between the 19^th^ of September and the 13^th^ of December 2021, severely damaged the flora and fauna within 2.5 km around the eruption [2]. The surrounding Canary Pine forests were significantly affected, which suffered chlorotic damage from exposure to volcanic gases [3,4]. In addition, large amounts of eruptive pyroclastic material were deposited (fine ashes and more coarse-grained particles, dependent on the intensity of and the distance to the eruption) as a tephra layer over a large part of the island (Fig. 1). Tephra deposits are known to affect all aspects of vegetation recovery after volcanic eruptions [5,6]. They can completely reset the seed bank already with approximately 10 cm thickness [7]. It is postulated that the depth of the tephra deposits functions as a selective evolutionary driver [8]. In addition, the tephra layer will likely affect the water cycle and nutrient availability in affected ecosystems and will leach volcanic compounds for many months and years [9,10]. We have, therefore, created an interpolated dataset of tephra depth all over La Palma, which will be crucial in successfully monitoring vegetation recovery in the coming years after the eruption.

## 3 Data Description

**Fig. 2:**
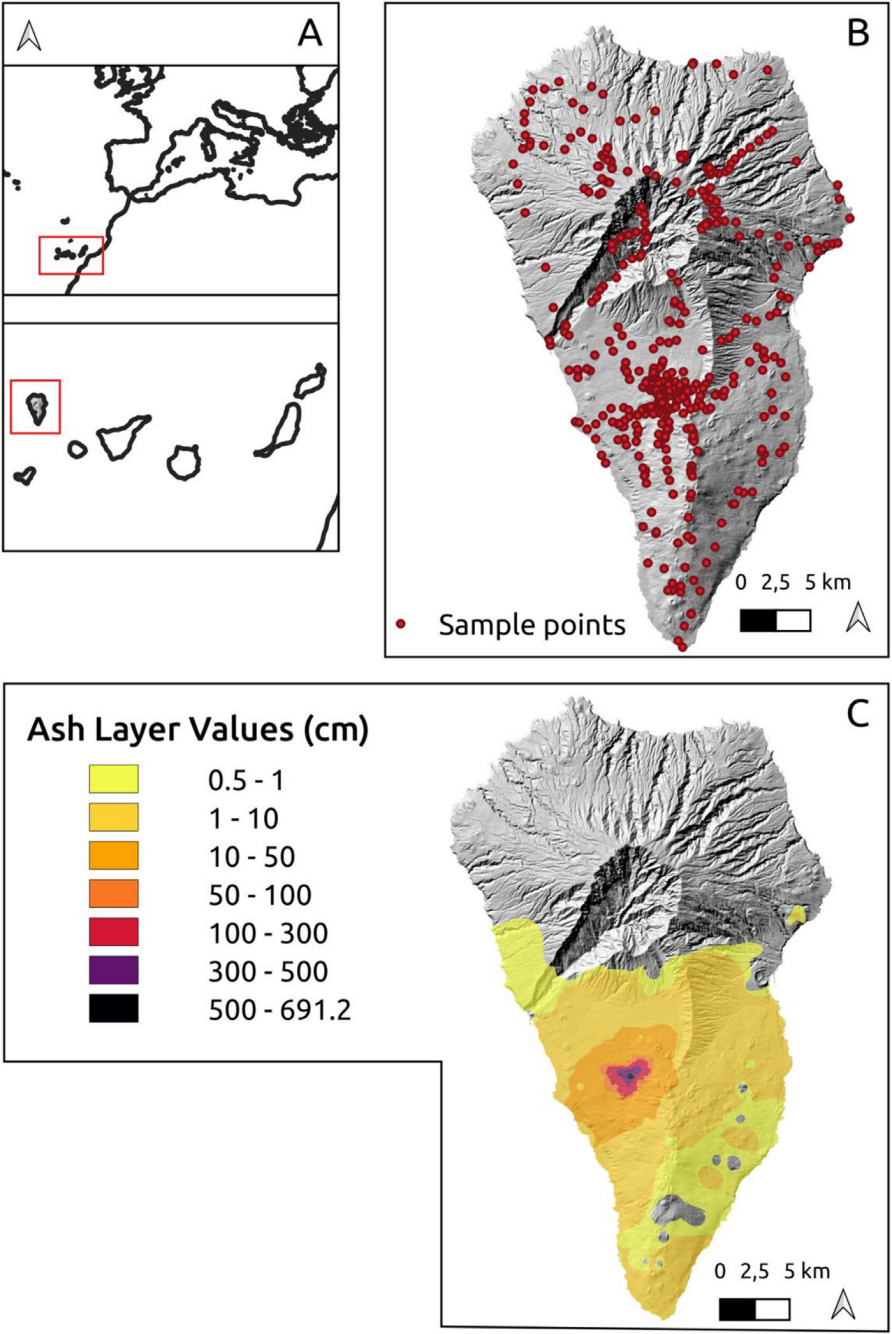
Map of the island of La Palma, Canary Islands, Spain, representing the shapefile and GeoTIFF files in the data repository. The shapefile is compiled from both field and drone-sourced tephra depth measurements (B). The shapefile sample points were used as input in the IDW interpolation (C). The colour scale indicates the interpolated tephra depth values ranging from shallow depths (yellow) to large depths (purple). The figure includes a Digital Elevation Model (DEM) at 2m resolution [12] which is not included in the repository. The reference location of the island of La Palma and the Canary Islands is displayed in Box A.

The dataset consists of two files: (1) a shapefile (with supporting .dbf, .shx, .qmd, .prj, and .cpg files) consisting of 481 total tephra depth measurements that were either recorded in the field (415 points) or extracted from the drone data (66 points) provided by Civico et al. [11]; (Fig. 2) (2) the output tephra interpolation raster file.

The shapefile's point measurements (field: “ash_field”) were used as input data for our IDW interpolation. The tephra interpolation raster file covers the full extent of La Palma's land surface at 2 m spatial resolution with ash depth values (in cm) ranging from 0 (no ash layer) to 691.219. The maximum value indicates the largest recorded measurement as the interpolation is confined to the minimum and maximum of the measured values. We then added the interpolated values to the point shapefile as an additional field (field: “ash_idw”). From here, we could assess the accuracy of the interpolation by calculating the RMSE. The RMSE of the interpolation is 0.34.

## 4 Experimental Design, Materials and Methods

### 4.1 Tephra layer field measurements

**Fig. 3:**
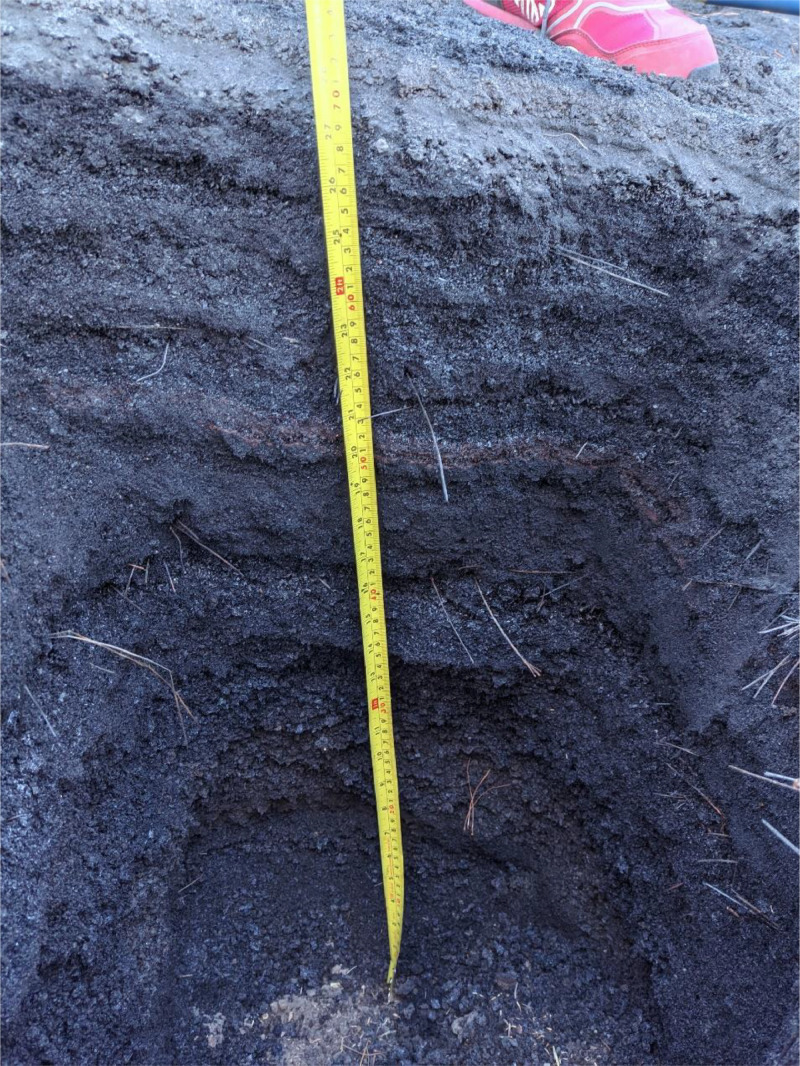
A sampling hole showing the layering of the tephra to a depth of 68 cm. Occasional needles from Canary Pine (*Pinus canariensis*) that were lost during the eruptive phase are present in the tephra. (Photo: Frank Weiser).

Tephra depth, as the total of all airborne pyroclastic sediments of different grain sizes, was measured at 415 sampling points distributed over the island (Fig. 2). Only undisturbed, flat surfaces were sampled. Holes were dug until a remarkable color change to the weathered (oxidized) previous soil surface was observed, or until bedrock was reached. Historic tephra layers, e.g. from the 1949 volcanic San Juan eruption, could also be distinguished due to a stark color difference due to weathering and oxidation as well as the presence of a subfossil organic litter layer (Fig. 3). Closer to the volcano, the tephra layer reached several meters of depth. Due to time and safety constraints, holes were dug to a maximum depth of 150 cm. To later improve interpolation accuracy, as many sampling holes were dug close to 150 cm depth as possible. Additional evidence due to construction works showed tephra layers of more than 3 m.

To fill in the gaps of our sampling close to the volcanic crater, where the tephra was too deep to sample, and to appropriately represent the thick tephra layer closer to the crater, two remote sensing data products were evaluated for their accuracy. Data points were then extracted from the more accurate of the two products. The first product used was elevation change data from Copernicus Emergency Management System (EMS) EMSN119 [13]. The data product uses a Spanish Lidar-based Digital Terrain Model (DTM) from 2015 [12] combined with optical Pléiades Tri-Stereo data to calculate a surface change layer for both the lava stream and crater surroundings. The RMSE given for elevation calculations of the Pléiades data used is 0.79 m, which is not accurate enough for the shallow tephra layers but sufficient for the massive tephra deposits around the craters. The second product evaluated was a Digital Surface Model (DSM) derived from over 12,000 drone photographs coupled with structure-from-motion photogrammetry [11] with a calculated RMSE of 0.26 m. A digital terrain model (DTM) with 2 m spatial resolution [12] was subtracted from the DSM provided by Civico et al. [11] to derive the surface change due to tephra deposition.

Both products show nearly identical surface changes over both the crater and the lava stream but differ up to several meters over the tephra-covered surroundings of the crater. The surface change values of the drone-derived model are in much better agreement with our field measurements and observations. It was suspected that the uniform surface and color of tephra-covered ground decreases the accuracy of Pléiades Tri-Stereo data in the areas around the craters and lava stream. Nonetheless, we elected the Civico et al. [11] data. The difference caused by tephra was extracted for 66 manually selected data points close to the crater, bringing the total number of samples to 481 points. The presence of *Pinus canariensis* individuals introduced errors in the DSM. Therefore, only data from forest gaps was used. In addition, while the overall accuracy of Civico et al. [11] was very high, only sites with a difference of at least 3 meters were selected to minimize errors introduced by methodological differences in the models.

### 4.2 Interpolation using IDW

An interpolation of the tephra layer data was performed at 2 m resolution using Inverse Distance Weighting (IDW) (Fig. 1). IDW is an exact deterministic interpolation method that predicts values of unknown points within the range of the measured values by averaging the weighted distance between sample points [14]. The interpolation was calculated using the Spatial Analyst toolbox in ArcMap 10.8.1 [15]. This toolbox was selected since our dataset was not large enough to warrant using other Esri toolboxes, such as the Geostatistical Analyst [16]. The dataset was compiled to later estimate the surface volume of the ash layer as a 2-dimensional layer, and thus the 3D Analyst toolbox was not necessary either. The parameters set for the IDW interpolation can be viewed in Table 1.

**Table 1:** Input parameters for the ArcMap IDW model.

Power	2
Output cell size	2
*Search neighborhood settings:*	
Search neighborhood type	Standard
Major/minor semiaxis	12161.79/12161.79
Max/minimum neighbors	15/10
Sector type	1
Angle	0
Weight field	None

Although geostatistical interpolation methods such as kriging are typically favored for spatial interpolations [17,18], IDW yielded a better RMSE value for our data (0.34) when compared with ordinary kriging (OK) in the same ESRI Spatial Analyst toolbox. Adopting more advanced variations of kriging, for example with external drift (KED) or gradient plus inverse distance squared (GIDS), could improve accuracy if parameterized appropriately [18]. However, the performance of an interpolation is often dependent on context and the accuracy metric utilized. Since spatial interpolations have been widely deployed in environmental sciences, they have diverse applications and are difficult to compare. For example, Li and Heap [18] compared the performance of over 70 spatial interpolation methods and sub-methods, including combined methods, across 80 environmental variables. They found that though kriging often performed better than non-geostatistical methods, factors such as sampling design may heavily impact performance. Given the high performance of the IDW interpolation of our in-situ tephra measurements, IDW was selected as a suitable interpolation method for our dataset.

IDW operates using a power parameter that raises the inverse of the distance to a mathematical power, thereby permitting control over the significance of measure values [16]. The higher the power parameter, the more significant the observed value becomes, and a coarser surface layer is achieved. A relatively gradual deposition of tephra was observed in the field and thus the power was limited to the default value of 2, which reflects a smoother surface in the resulting product. This is deemed more realistic given that in-situ sampling occurred several months following the end of the volcanic eruption, where wind and runoff may have neutralized any marginal differences in the surface profile during the time of sampling. The IDW input parameters in Table 1 were selected based on performance when compared to other IDW model runs. Interpolation performance was assessed by how well the model agreed with what was observed during the field campaign. Despite running the model several times with adjusted parameters, such as the power, search neighborhood type, number of neighbors and sector type, the default parameter settings were the most optimal for our input dataset. The only parameter that was not a default value was the output cell size (2 m).

## Limitations

Not applicable.

## Ethics Statement

The authors have read and followed the ethical requirements for publication in Data in Brief and confirmed that the current work does not involve human subjects, animal experiments, or any data collected from social media platforms.

## CRediT authorship contribution statement

**Christopher Shatto:** Investigation, Methodology, Software, Visualization, Writing – original draft. **Frank Weiser:** Conceptualization, Investigation, Methodology, Visualization, Writing – original draft. **Anna Walentowitz:** Conceptualization, Investigation, Methodology, Writing – review & editing. **Reinhold Stahlmann:** Validation, Writing – review & editing. **Samip Shrestha:** Methodology, Writing – review & editing. **María Guerrero-Campos:** Writing – review & editing. **Félix Manuel Medina:** Writing – review & editing. **Manuel Nogales:** Writing – review & editing. **Anke Jentsch:** Funding acquisition, Writing – review & editing. **Carl Beierkuhnlein:** Conceptualization, Funding acquisition, Supervision, Writing – review & editing.

## Data Availability

IDW interpolation dataset of ash and tephra deposition following the 2021 Tajogaite volcanic eruption on La Palma, Canary Islands, Spain (Original data) (Zenodo).
